# Icariside II Restores Vascular Smooth Muscle Cell Contractile Phenotype by Enhancing the Focal Adhesion Signaling Pathway in the Rat Vascular Remodeling Model

**DOI:** 10.3389/fphar.2022.897615

**Published:** 2022-06-13

**Authors:** Junyuan Lv, Xintong Li, Hongyu Wu, Jiayang Li, Boyang Luan, Yiqi Li, Yeli Li, Danli Yang, Hao Wen

**Affiliations:** ^1^ Breast and Thyroid Surgery, Department of General Surgery, The Affiliated Hospital of Zunyi Medical University, Zunyi, China; ^2^ Department of Vascular Surgery, The First Affiliated Hospital of China Medical University, Shenyang, China; ^3^ Key Laboratory of Basic Pharmacology of Ministry of Education and Joint International Research Laboratory of Ethnomedicine of Ministry of Education, Zunyi Medical University, Zunyi, China; ^4^ Drug Clinical Trial Institution, The Affiliated Hospital of Zunyi Medical University, Zunyi, China; ^5^ Department of Trauma Center, The First Affiliated Hospital of China Medical University, Shenyang, China

**Keywords:** icariside II, vascular smooth muscle cell, phenotypic transition, vascular remodeling, herbal medicine

## Abstract

Vascular smooth muscle cell (VSMC) phenotypic transition represents the fundamental pathophysiological alteration in the vascular remodeling process during the initiation and progression of cardiovascular diseases. Recent studies have revealed that Icariside II (ICS-II), a flavonol glycoside derived from the traditional Chinese medicine Herba Epimedii, exhibited therapeutic effects in various cardiovascular diseases. However, the therapeutic efficacy and underlying mechanisms of ICS-II regarding VSMC phenotypic transition were unknown. In this study, we investigated the therapeutic effects of ICS-Ⅱ on vascular remodeling with a rat’s balloon injury model *in vivo*. The label-free proteomic analysis was further implemented to identify the differentially expressed proteins (DEPs) after ICS-II intervention. Gene ontology and the pathway enrichment analysis were performed based on DEPs. Moreover, platelet-derived growth factor (PDGF-BB)-induced primary rat VSMC was implemented to verify the restoration effects of ICS-II on the VSMC contractile phenotype. Results showed that ICS-II could effectively attenuate the vascular remodeling process, promote SMA-α protein expression, and inhibit OPN expression *in vivo*. The proteomic analysis identified 145 differentially expressed proteins after ICS-II intervention. Further, the bioinformatics analysis indicated that the focal adhesion signaling pathway was enriched in the ICS-II group. *In vitro* studies showed that ICS-II suppressed VSMC proliferation and migration, and promoted VSMC contractile phenotype by modulating the focal adhesion signaling pathway. Taken together, our results suggest that ICS-II attenuates the vascular remodeling process and restores the VSMC contractile phenotype by promoting the focal adhesion pathway.

## Introduction

Cardiovascular diseases (CVD) and associated co-morbidities are the leading cause of death globally (Roth et al., 2020). Many of these pathologies such as hypertension (Renna et al., 2013), atherosclerosis (Kiechl and Willeit, 1999), stenosis (Pasterkamp et al., 2000), and aneurysms (Frösen et al., 2019; Cañes et al., 2021) are accompanied by sophisticated pathophysiological alterations of the vascular tone and structure, in response to external stimuli as well as genetic preconditioning, which is termed as vascular remodeling (Lyle and Taylor, 2019). The phenotypic transition of vascular smooth muscle cells (VSMCs) in the media, the most dominant cell type in vessel wall composition, plays an essential role in vascular remodeling (Jaminon et al., 2019; Shi et al., 2019). Thereby, medical treatments targeting the VSMC phenotypic transition are crucial for overcoming the tremendous burden caused by vascular remodeling of CVDs on human health (Chakraborty et al., 2021).

To date, several pharmaceuticals targeting VSMC phenotypic transition, including rapamycin and paclitaxel, have been applied in clinical practice to reverse the vascular remodeling process (Briguori et al., 2021). In addition, recently, herbal medicines, especially derivatives from anti-atherosclerotic plants, are raising attention for modulating the VSMC phenotypic transition (Saleh Al-Shehabi et al., 2016). Icariside II (ICS-II, C27H30O10, 514.57 g/mol), the bioactive metabolic ingredients of the traditional Chinese medicine Herba Epimedii, have been reported to exhibit a wide range of biological and pharmacological properties including anti-inflammatory, anti-apoptotic, and anti-cancer properties (Zheng et al., 2020). Furthermore, substantial investigations revealed the valid therapeutic efficacy of ICS-II in various cardiovascular diseases (Qi et al., 2017; Liu et al., 2018; Guan et al., 2020). However, evidence about ICS-II in the VSMC phenotypic modulation is still limited and needs to be further elucidated.

Thus, the present study aims to investigate the pharmacological effects of ICS-II on the VSMC phenotypic transition and reveal the underlying mechanisms through *in vivo* and *in vitro* experiments. Our results highlight the therapeutic benefits of ICS-II targeting VSMC phenotypic transition and support the potential of pharmacological strategies in CVD management.

## Materials and Methods

### Ethics

The protocols and procedures in this present study were approved by the Experimental Ethics Committee of Zunyi Medical University. All protocols were consistent with the Guide for the Care and Use of Experimental Animals at the Animal Center of Zunyi Medical University.

### Study Design and Animal Model Induction

A total of 30 SPF (specific pathogen-free) male Sprague–Dawley (SD) rats (body weight:300–400 g) were used in this study. The rats were randomly divided into three groups and subjected to the following treatments: the sham group (*n* = 10): received a sham operation with the same volume of saline by gavage daily; the control group (*n* = 10): model induction with the same volume of saline by gavage daily; the ICS-II group (*n* = 10): model induction with ICS-II 20 mg/kg by gavage daily. The protocols for the rat carotid artery balloon injury model were described previously (Lv et al., 2018). Briefly, the rats were anesthetized with 5% isoflurane in oxygen. A midline cervical incision was made to expose the bilateral carotid arteries. A 2F Fogarty balloon catheter (Edwards Lifesciences Corporation; United States) was inserted into the left common carotid artery (CCA) three times to disrupt the endothelium through the external carotid artery (ECA). After that, the ECA was sutured and the left CCA was re-perfused. All processes during the operation were performed by a single researcher. All rats were euthanatized on day 14 for the following experiments.

### Morphometric Analysis

For the morphometric analysis, artery samples were fixed in 4% paraformaldehyde, embedded in paraffin, and then sectioned into 4 mm thickness. After deparaffinization and rehydration, sections were stained with hematoxylin and eosin (H&E) to observe the morphological changes. For each section, five randomly selected non-continuous microscopic fields were pictured to determine the mean thickness value of the intima and media, respectively. Finally, the thickness ratio of intima to media of each section was determined.

### Immunohistochemistry

IHC experiments were implemented for assessing the protein expression level of the VSMC synthetic marker OPN *in vitro*. Protocols for the IHC experiments were described previously (Wen et al., 2020). In brief, after de-paraffinization and rehydration, sections were incubated with 3% H_2_O_2_ to deactivate endogenous peroxidase followed by a heat-induced antigen retrieval process. Then, sections were incubated with primary and secondary antibodies before image acquisition.

### Tissue Lysis and Protein Extraction

The arterial tissue samples were lysed with an SDT (4% SDS in 0.1 M Tris-HCl, pH = 7.6) solution, transferred into a Lysing Matrix A (MP, cat: 6910-100-99219) tube, and homogenized and broken with an MP homogenizer (MP Fastprep-24 5G, 24 × 2, 6.0 M/S, 60 s, twice). The supernatant was filtered through a 0.22 µm centrifuge tube and the filtrate was collected. Protein quantification was performed using the BCA (Beyotime Biotechnology, P0012) method. About 80 μg of the protein solution was taken for each sample, and DTT (dithiothreitol, Sigma, 43819-5G) was added to a final concentration of 100 mM, boiled for 5 min, and cooled to room temperature. Then, 200 μL of the UA (8 M urea in 0.15 M Tris-HCl, pH = 8.5) buffer was added, mixed, and transferred to a 30 kDa ultracentrifuge tube (Sartorius, VN01H22); the mixture was centrifuged at 12,500 g for 15 min, and the filtrate (repeat this step once) was discarded. Further, 100 μL of IAA (iodoacetamide, Sigma, I1149-5G) buffer (100 mM IAA in UA) was added and the mixture was shaken at 600 rpm for 1 min and centrifuged at room temperature for 30 min at 12,500 g for 15 min. Then, 100 μL of the UA buffer was added and centrifuged at 12,500 g for 15 min. Subsequently, 100 μL of 40 mM NH_4_HCO_3_ solution was added, centrifuged at 12,500 g for 15 min, and the procedure was repeated twice. After that, 40 μL of trypsin buffer (4 μg of trypsin in 40 μL of 40 mM NH_4_HCO_3_ solution) was added and the mixture was shaken at 600 rpm for 1 min, and allowed to stand at 37°C for 16–18 h. The collection tube was replaced with a new one and centrifuged at 12,500 g for 15 min; then 20 μL of 40 mM NH_4_HCO_3_ solution was added and centrifuged at 12,500 g for 15 min, and the filtrate was collected. The peptide was desalted by C18 Cartridge (Waters, WAT023590), and 40 μL of 0.1% formic acid (Thermo Fisher Scientific, A117) solution was added after the peptide was lyophilized; the peptide was quantified (OD 280).

### Label-free Quantitative Proteomic Analysis

For LC–MS/MS analysis, each sample was analyzed using the Easy nLC system (Thermo Fisher Scientific). Buffer solution A was 0.1% formic acid in water and solution B was 0.1% formic acid with 80% acetonitrile in water. The chromatographic column was equilibrated with 100% solution A. Samples were separated by autosampler loading onto an analytical column (Thermo Fisher Scientific, Acclaim PepMap RSLC 50 μm × 15 cm, nano Viper, P/N164943) at a flow rate of 300 nL/min. The samples were separated by chromatography and analyzed by mass spectrometry using a Q Exactive (Thermo Fisher Scientific) mass spectrometer. Precursor mass spectra were recorded in a 350–1,800*m*/*z* (mass/charge ratio) mass range at 70,000 resolution and 17,500 resolution for fragment ions. Data obtained were analyzed using MaxQuant (version 1.5.5.1) against the UniProt database (Uniprot_RattusNorvegicus_36080_20180123) based on the LFQ (label-free quantitation) method (Cox et al., 2014) with the following parameters: enzyme, trypsin; max missed cleavages, two; main search, 4.5 ppm; first search, 20 pp; MS/MS tolerance, 20 ppm; fixed modification, carbamidomethyl (C); dynamic modification, oxidation (M), acetyl (protein N-term); peptide and protein FDR (false discovery rate), <0.01.

### Analysis of Differentially Expressed Proteins (DEPs)

The DEP Package (Zhang et al., 2018) in R software (version: 4.0.2) was applied for differentially expressed protein analysis. Proteins were filtered to remove contaminants and missing values in more than one sample per group. Then, background correction and normalization by the variance-stabilizing transformations method (Huber et al., 2002) were performed. The remaining missing values were estimated and imputed by impute.MinProb function for left-censored missing data (Lazar, 2015). A differential expression analysis based on protein-wise linear models with empirical Bayes statistics was then performed on the imputed dataset. Proteins with the absolute value of fold change >1.5 and *p*-value < 0.05 were identified as significant DEPs.

### Construction of Protein–Protein Interaction (PPI) Network

Construction of the PPI network was implemented with the Search Tool for Retrieval of Interacting Genes/Proteins (STRING: https://string-db.org/) database (Szklarczyk et al., 2019) with criteria of medium confidence (interaction score >0.4). Then, the derived PPI network was visualized and analyzed by the analysis network function via Cytoscape (version 3.8.0) (Shannon et al., 2003) software. Proteins in the aforementioned PPI network with a degree >5 were selected for visualization and subsequent enrichment analysis.

### Functional and Pathway Enrichment Analysis

Enrichment analysis regarding Gene Ontology (GO) (Ashburner et al., 2000) terms describing the biological process (BP), molecular function (MF), and cellular component (CC), as well as the Kyoto Encyclopedia of Genes and Genomes (KEGG) (Ogata et al., 1999) pathway, were performed by using the clusterProfiler (Yu et al., 2012) package in R. Enriched terms with FDR <0.05 were selected and visualized.

### Cell Culture and Experimental Design

Rat VSMCs were primarily isolated and cultured by the explant method as described previously (Li et al., 2020). In brief, thoracic aortas were gently dissected from 8-week-old male SD rats and the surrounding connective tissue was removed. Aortas were longitudinally dissected, and the epithelial monolayer was detached by scraping. Tissue fragments were delivered to culture plates with Dulbecco’s modified Eagle’s medium (DMEM) supplemented with 10% fetal bovine serum (FBS) and 1% penicillin/streptomycin. After 2 days, tissue fragments were removed and the sprouting VSMCs were maintained until 80% confluence. Cells were trypsinized and seeded on a culture flask for *in vitro* experiments. All cells were maintained in a humidified atmosphere containing 5% CO_2_ at 37°C, and passages between three and six were used in the present study.

The primarily cultured VSMCs were randomly divided into three experimental groups: the control group, 0.5% DMSO; the PDGF group, 25 ng/ml PDGF; and the ICS-II group, 20 μM ICS-II + 25 ng/ml PDGF. All experimental cell groups were incubated with corresponding drugs for 24 h, respectively.

### Immunofluorescence (IF)

Briefly, cells were seeded onto coverslips and incubated for 24 h in 6-well plates. After that, the cells were fixed with formaldehyde and permeabilized with 0.25% Triton X-100 in PBS and 1% bovine serum albumin (BSA) for 5 min. Cells were then blocked with 5% goat serum in PBS for 1 h. Then, the cells were incubated with primary antibodies overnight at 4°C and treated with secondary antibodies in 3% BSA in PBS for 2.5 h at room temperature. Then, the nuclei were stained with 4′,6-diamidino-2-phenylindole (DAPI) and observed under a fluorescence microscope. Negative isotypes were incubated with PBS under the same conditions.

### Western Blot

Briefly, the total proteins from different groups were extracted by lysing VSMCs with RIPA buffer (Beyotime, Shanghai, China), and the protein concentration was determined by using a BCA Protein Assay Kit (Beyotime, Shanghai, China). Then, the protein samples were separated by SDS–PAGE and subsequently transferred to PVDF membranes (Millipore, United States). After being blocked in 5% BSA at room temperature for 2 h, membranes were incubated for 12 h at 4°C with primary antibodies. After being washed three times with PBS, HRP-conjugated secondary antibodies were used to incubate the membranes for 1 h at room temperature. Finally, protein bands were visualized using chemiluminescence equipment.

### *In vitro* Scratch Assay

The primarily cultured VSMCs were seeded into 6-well plates at a cell density of 5×10^5^ until 80% confluence. The cells were scratched with a 10 μL pipette tip, the residue was rinsed, and the corresponding drugs with the medium containing 1% serum were added. The cell scratch assay was observed under an inverted microscope (Olympus, Japan), and photographed at 0 and 24 h, respectively.

### Transwell Migration Assay

The VSMC transwell migration assay was implemented using 24-well transwell chambers (8 μm pore size; BD Biosciences, United States). Briefly, a total of 10,000 cells per well were re-suspended in a serum-free medium with corresponding interventions, respectively. Cells were then added into the top chamber with the same medium containing 10% FBS at the bottom of the chambers. After 24 h, the migrated cells through the biofilms were fixed and stained with a crystal violet staining solution (C0121, Beyotime, China) for morphological observation with the microscope (Olympus, Japan).

### Statistical Analysis

Results are expressed as mean ± SEM (standard error of the mean) and the statistical significance between the means of the two groups was determined by the two-tailed *t*-test or Mann–Whitney test. The *p*-value < 0.05 was considered statistically significant. GraphPad Prism (version 8.2.1, United States) was used for all statistical analyses.

## Results

### ICS-II Attenuated Vascular Remodeling and Restored VSMC Contractile Phenotype *in vivo*

**FIGURE 1:**
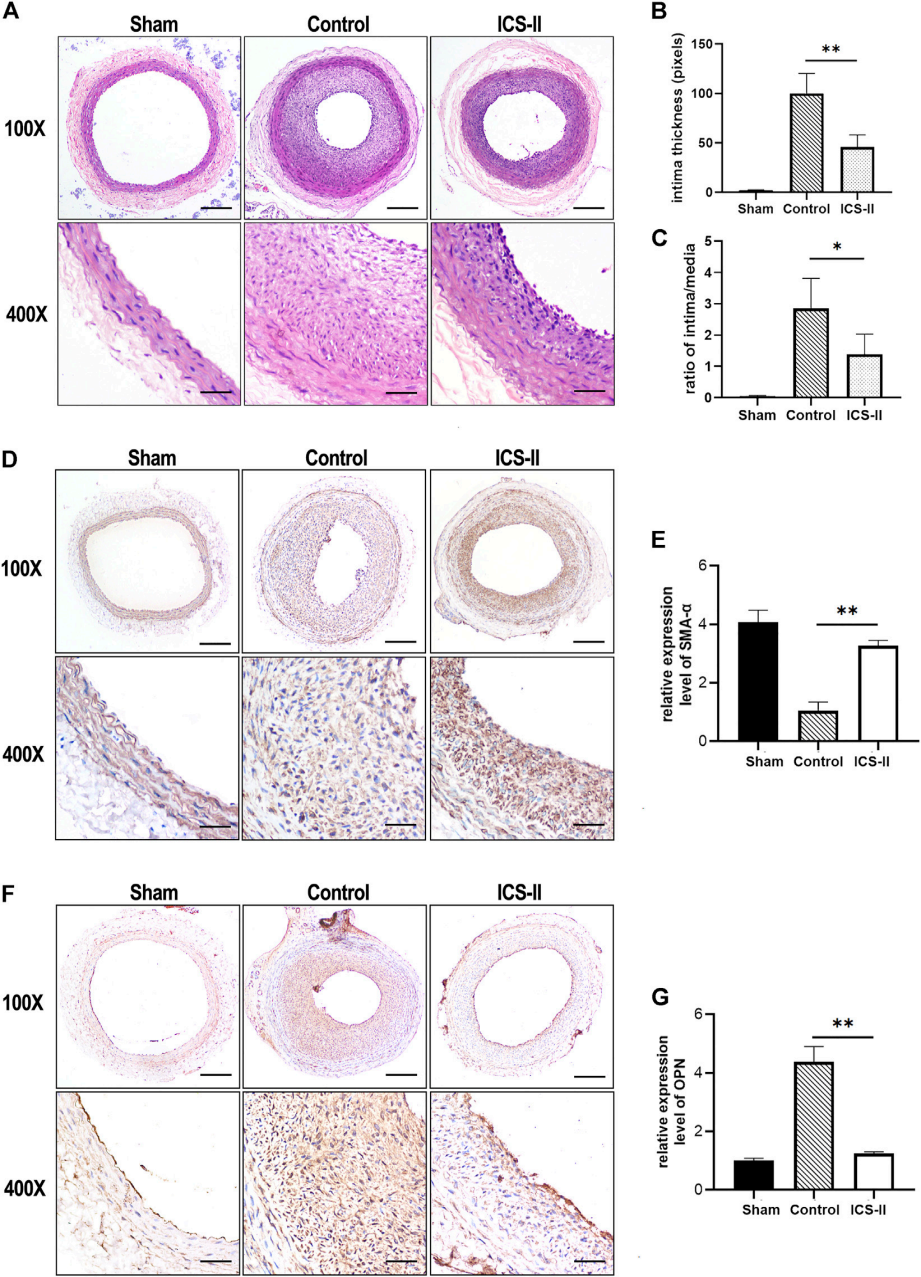
ICS-II attenuated vascular remodeling and restored the VSMC contractile phenotype in vivo. **(A)** Representative H&E staining images of the injured left common carotid arteries from the sham group, control group, and ICS-II group, respectively. **(B)** Quantification of the intima thickness. **(C)** The lesion was calculated using the ratio of intima/media. **(D)** Representative IHC images of SMA-α in the sham group, control group, and ICS-II group, respectively. **(E)** Quantification of the SMA-α expression level in vivo. **(F)** Representative IHC images of OPN in the sham group, control group, and ICS-II group, respectively. **(G)** Quantification of the OPN expression level in vivo. X100, scale bar = 100 μm; X400, scale bar = 20 μm; Data are presented as mean ± S.E.M. N = 5 per group. ^*^ *p* < 0.05, ^**^ *p* < 0.01, ^***^ *p* < 0.001, ^****^ *p* < 0.0001.

To investigate the role of ICS-II in vascular remodeling, a rat carotid artery balloon injury model was implemented, which is capable of mimicking vascular remodeling as well as VSMC phenotypic transition validly *in vivo* (Tulis, 2007; Zhang and Trebak, 2014). Results from the morphometric analysis showed that ICS-II significantly suppressed intima thickness and intima/media ratio (Figure 1A–C) at day 14 compared to the control group (*p* < 0.05). Immunohistochemistry experiments showed that SMA-α, a contractile phenotype marker of VSMC, was significantly enhanced in the arterial tissue compared with the control group (*p* < 0.05), as shown in Figures 1D,E. In contrast, the expression level of synthetic marker OPN was attenuated after ICS-II administration (Figure 1F,G). The data aforementioned indicated that ICS-II effectively attenuated the vascular remodeling process and restored VSMC contractile phenotype *in vivo*.

### DEP Analysis After ICS-II Intervention

**FIGURE 2:**
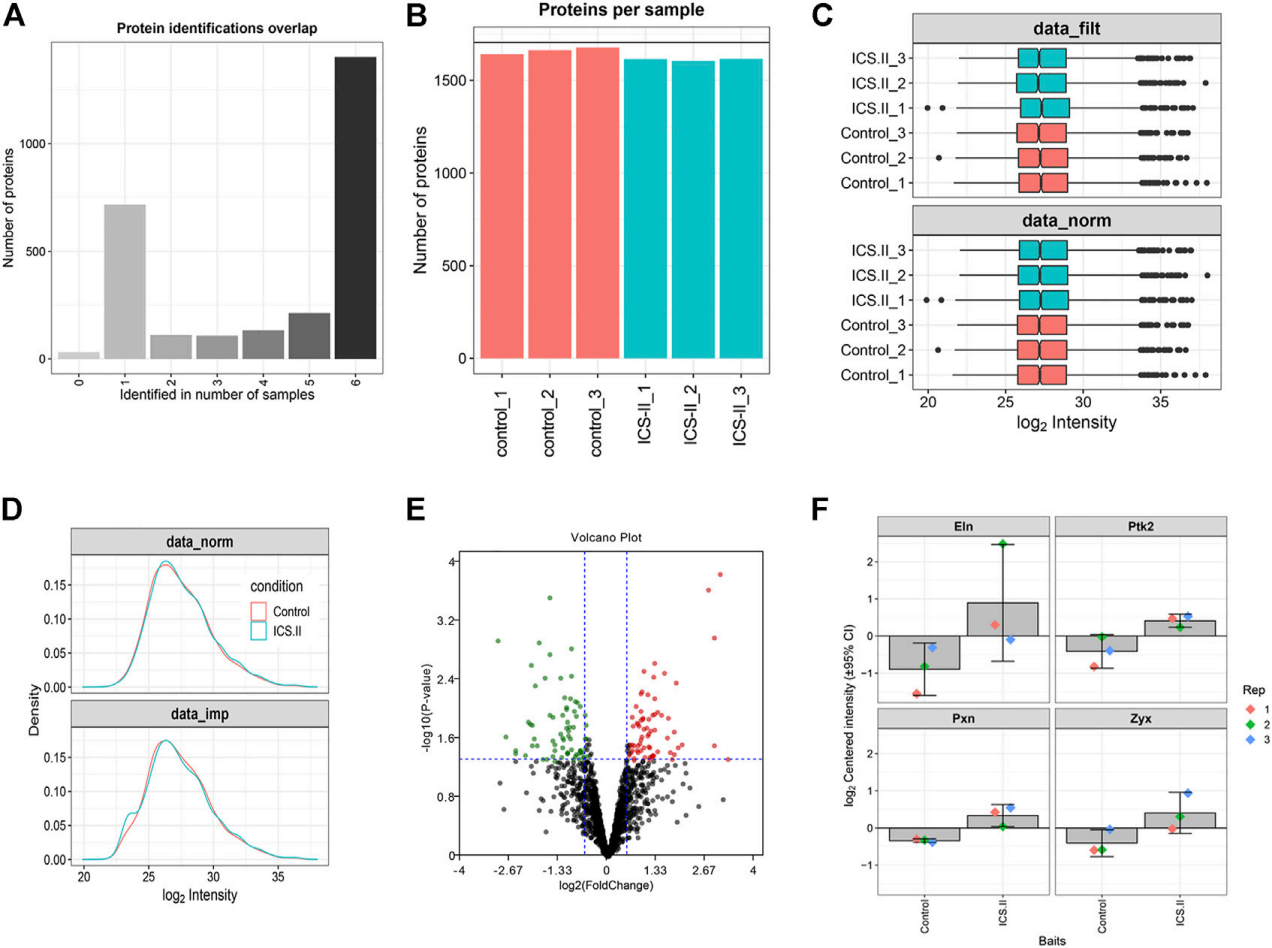
Differentially expressed protein (DEP) analysis after ICS-II intervention. **(A)** Overlap of protein identifications between samples. **(B)** The number of identified proteins per sample. **(C)** Comparison of raw and normalized data. **(D)** Protein intensity distributions before and after missing value imputation. **(E)** Volcano plot for DEPs between the ICS-II group and control group. **(F)** Bar plots of ELN, PTK2, PXN, and ZYX expression levels. ELN: elastin; PTK2: protein tyrosine kinase 2; PXN: paxilline; ZYX: Zyxin.

To further elucidate the proteomic changes after ICS-II intervention in vascular remodeling, the label-free quantitative proteomic analysis was implemented. With the steps of filtering, normalization, and imputing missing values embedded in the DEP algorithm (Figure 2A–D), a total of 145 differential proteins (including 70 up-regulated proteins and 75 down-regulated proteins) in the ICS-II group compared to the control group, were identified. The volcano plot of the differential analysis is presented in Figure 2E. In addition, the expression levels of PTK2, PXN, ZYX, and ELN are plotted in Figure 2F, respectively. Results indicated significantly higher protein expression levels of PTK2, PXN, ZYX, and ELN in the ICS-II group compared to the control group (*p* < 0.05).

### Bioinformatic Analysis Indicated ICS-II Enhanced VSMC Contractile Phenotype Through the Focal Adhesion Pathway

**FIGURE 3:**
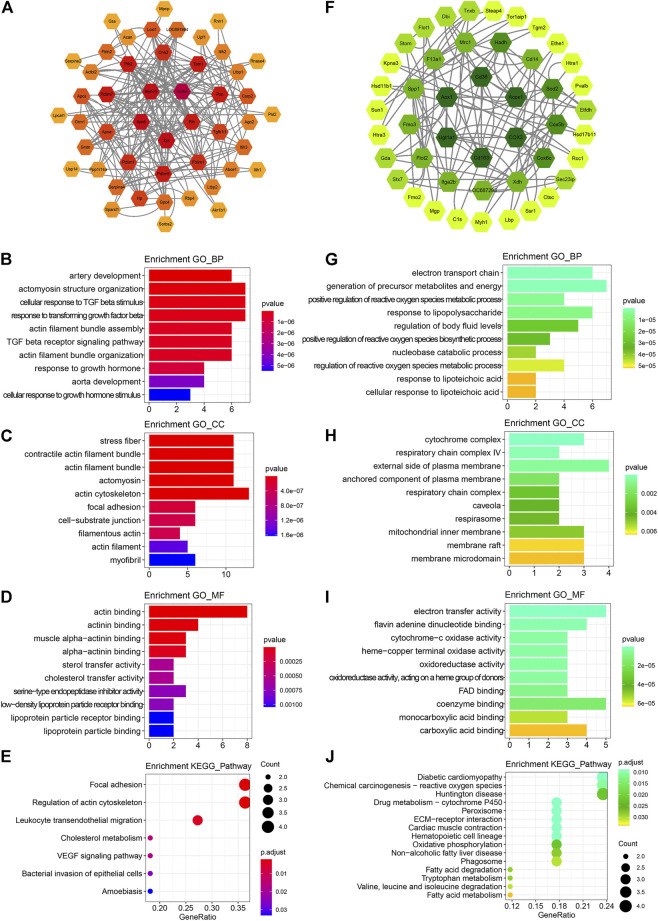
PPI network construction and functional annotation. **(A)** PPI network construction, **(B)** biological process, **(C)** cellular component, **(D)** molecular function, and **(E)** KEGG pathway enrichment analysis based on 70 up-regulated DEPs, respectively. **(F)** PPI network construction, **(G)** biological process, **(H)** cellular component, **(I)** molecular function, and **(J)** KEGG pathway enrichment analysis based on 75 down-regulated DEPs. DEPs: differentially expressed proteins; PPI: protein–protein interaction; BP: biological process; CC: cellular component; MF: molecular function; KEGG: Kyoto Encyclopedia of Genes and Genomes.

To further elucidate the underlying protein–protein interactions and intricated networks, the PPI network, and enrichment analyses were conducted. Seventy up-regulated and 75 down-regulated proteins in the ICS-II group were imported into the STRING database to derive the PPI network, as shown in Figure 3A,F, respectively. Proteins with degrees >5 were further selected for the enrichment analysis. The biological process enrichment analysis showed that up-regulated DEPs were mainly enriched in the artery development, actomyosin structure organization, response to transforming growth factor β, actin filament bundle assembly, and organization. (Figure 3B). The cellular components enrichment analysis showed that up-regulated DEPs were mainly enriched in the stress fiber, contractile actin filament bundle, actin filament, focal adhesion, actin cytoskeleton, myofibril, etc. (Figure 3C). For molecular function, enriched terms mainly included actin binding, actinin binding, muscle alpha-actinin binding, etc. (Figure 3D). The KEGG pathway enrichment analysis revealed that up-regulated DEPs were mainly correlated with focal adhesion, regulation of actin cytoskeleton, VEGF pathway, etc. (Figure 3E). For down-regulated DEPs, enriched terms mainly included fatty acid metabolism, respiratory chain complex, regulation of reactive oxygen species biosynthetic and metabolic processes, amino acid metabolism, etc. (Figure 3G–J). The aforementioned bioinformatic results indicated that ICS-II attenuated vascular remodeling through the focal adhesion pathway by enhancing the VSMC contractile phenotype and suppressing signaling pathways related to cellular metabolism.

### ICS-II Inhibited VSMC Proliferation and Migration

**FIGURE 4:**
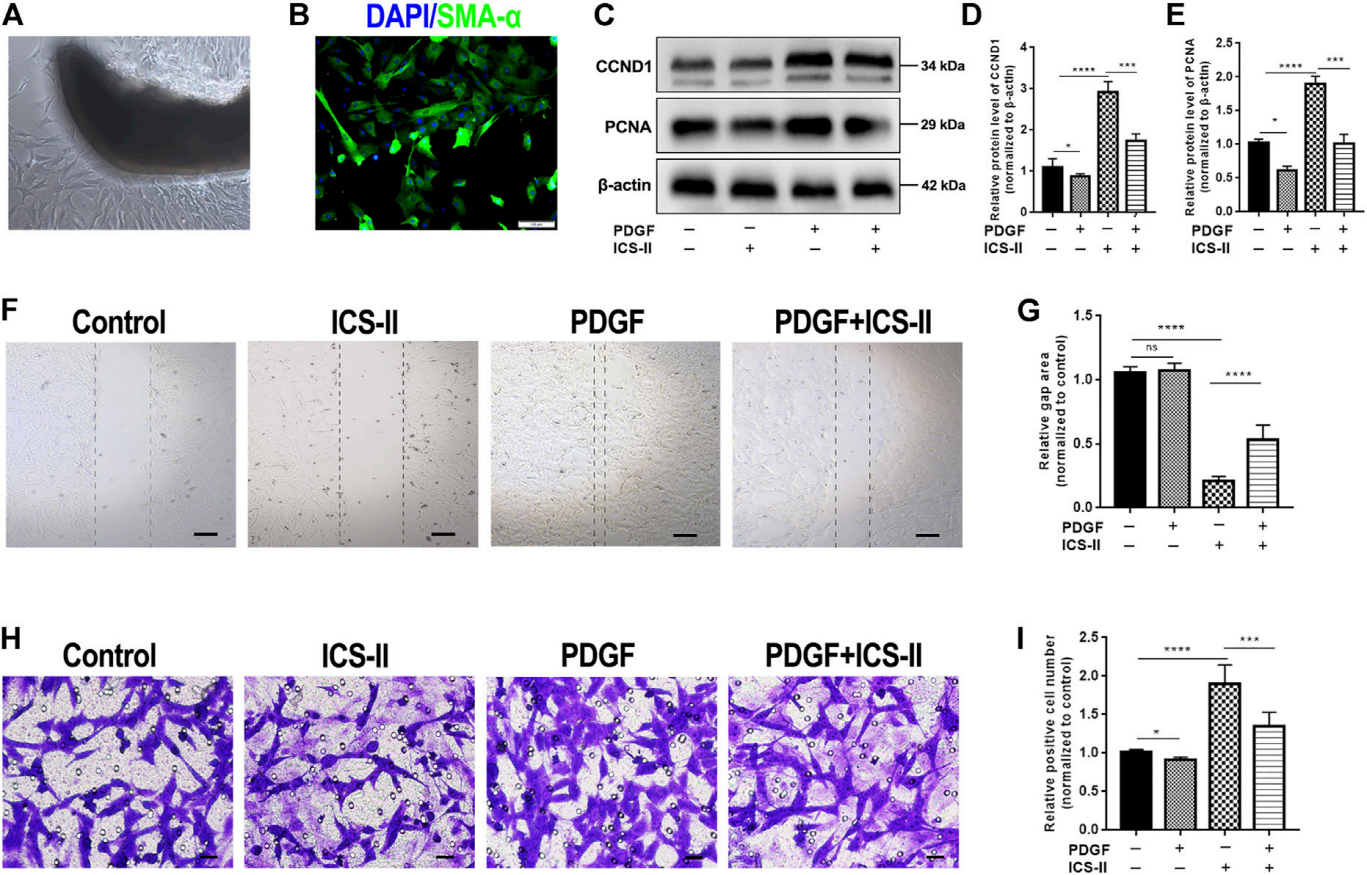
ICS-II inhibited VSMC proliferation and migration. **(A)** VSMCs were primarily cultured by using the explant method. **(B)** Verification of VSMCs at passage 3 with SMA-α (green) and DAPI (blue) staining. **(C)** Western blot images for PCNA and CCND1. Quantification of **(D)** CCND1 and **(E)** PCNA protein levels, respectively. **(F)** *In vitro* scratch assay for control, ICS-II and PDGF-BB, and PDGF-BB + ICS-II groups, respectively. **(G)** Quantification of the gap area in the scratch assay. **(H)** Transwell migration assay for control, ICS-II, and PDGF-BB and PDGF-BB + ICS-II groups, respectively. **(I)** Quantification of migrated cell number in the transwell assay. Scale bar = 20 μm; Data are presented as mean ± S.E.M. *N* = 5 per group. ^*^ *p* < 0.05, ^**^ *p* < 0.01, ^***^ *p* < 0.001, ^****^ *p* < 0.0001.

To further verify that ICS-II restored the VSMC contractile phenotype during vascular remodeling, we used primarily cultured rat aortic VSMC to conduct *in vitro* experiments (Figure 4A,B). Excessive proliferation is the major feature during VSMC phenotypic modulation. Western blot results showed that ICS-II significantly attenuated the protein expression levels of PCNA and CCND1 (Figure 4C–E), which indicated that ICS-II inhibited VSMC proliferation. Cell migration represents another essential hallmark for the VSMC phenotypic transition. Therefore, *in vitro* scratch assay and transwell assay were implemented to assess the ability of VSMC migration after ICS-II intervention. Results of the scratch assay revealed the suppressed migration ability of VSMC after ICS-II intervention compared to the PDGF-BB group (*p* < 0.05, Figure 4F,G). The transwell assay showed similar results, as shown in Figure 4H,I.

### ICS-II Restored VSMC Contractile Phenotype Through the Focal Adhesion Signaling Pathway

**FIGURE 5:**
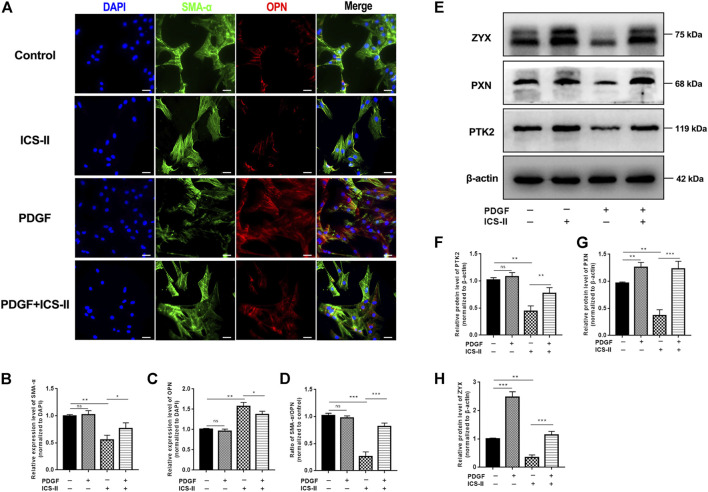
ICS-II restored VSMC contractile phenotype through the focal adhesion signaling pathway. **(A)** Co-staining of SMA-α (green) and OPN (red) for control, ICS-II, and PDGF-BB and PDGF-BB + ICS-II groups, respectively. Quantification of the relative expression level of **(B)** SMA-α, **(C)** OPN, and **(D)** ratio of SMA-α/OPN, respectively. **(E)** Western blot images for PTK2, PXN, and ZYX. Quantification of **(F)** PTK2, **(G)** PXN, and **(H)** ZYX protein levels, respectively. Scale bar = 20 μm; Data are presented as mean ± S.E.M. *N* = 5 per group. ^*^ *p* < 0.05, ^**^ *p* < 0.01, ^***^ *p* < 0.001, ^****^ *p* < 0.0001.

We next examined the protein expression level of SMA-α and OPN by cellular IF experiments. Results revealed that the ICS-II intervention significantly enhanced the SMA-α expression level while it inhibited the OPN protein expression compared to the PDGF-BB group (*p* < 0.05, Figures 5A–D). The aforementioned results showed that ICS-II restored the VSMC contractile phenotype *in vitro*.

To elucidate the role of the focal adhesion pathway in the ICS-II-induced VSMC contractile phenotype, we next examined the protein expression levels of PTK2, PXN, and ZYX after ICS-II intervention. Western blot showed significantly enhanced PTK2, PXN, and ZYX protein levels in the ICS-II group compared to PDGF-BB stimulation (*p* < 0.05, Figures 5E–H). Taken together, ICS-II restored VSMC contractile phenotype by enhancing focal adhesion signaling activity.

## Discussion

The vascular remodeling process mediated by VSMC phenotypic transition is the major pathophysiological basis of numerous cardiovascular diseases (Lyle and Taylor, 2019). Pharmacological inhibition of the VSMC phenotypic transition will inevitably provide solid strategies for the prevention and treatment of cardiovascular diseases. Emerging basic pharmacological studies underscored the vital implication of traditional Chinese herbal medicine for cardiovascular diseases and vascular remodeling processes (Liu and Huang, 2016; Wang et al., 2020). Herein, ICS-II, a bioactive flavonol glycoside derived from the traditional Chinese medicine Herba Epimedii, was validated for its therapeutic value in the vascular remodeling process. Results indicated that ICS-II attenuated vascular remodeling by restoring the VSMC contractile phenotype through the focal adhesion pathway.

The rat carotid artery balloon injury model is one of the most well-characterized and frequently practiced rodent models for investigating vascular remodeling (Holt and Tulis, 2013). Pathophysiological responses to this model include VSMC dedifferentiation into synthetic phenotype featured by VSMC proliferation and migration, enhanced extracellular matrix synthesis and deposition, and proliferation of endothelium and luminal narrowing. Deciphering the mechanisms of VSMC phenotypic regulation and elucidating how these mechanisms can be targeted for treatment are fundamental for cardiovascular diseases (Chakraborty et al., 2021). In this study, a rat carotid artery balloon injury model was selected for the therapeutic efficacy of ICS-II in vascular remodeling. The morphometric analysis revealed that ICS-II significantly suppressed the vascular remodeling process *in vivo*. Moreover, *in vitro* studies using PDGF-BB-stimulated VSMC further indicated that ICS-II restored VSMC contractile phenotype.

Maintenance of three-dimensional cellular integrity is crucial for VSMC contraction and preservation of the contractile phenotype (Yamin and Morgan, 2012). The major intracellular cytoskeletal components for contractile units of VSMC include actin, myosin, microtubules, and filaments. Moreover, the physical interactions between VSMC contractile units and the extracellular matrix also contribute to VSMC contractile capability, which is termed focal adhesion (Zhang and Gunst, 2008). Focal adhesions are integrin-mediated multi-protein assemblies that provide mechanical bridges between the intracellular actin skeleton and the extracellular matrix in numerous cell types (Geiger et al., 2009). Emerging studies have highlighted the fundamental role of the focal adhesion signaling pathway, beyond the classical calcium-dependent pathways, in modulating VSMC contraction and phenotypic alteration (Ribeiro-Silva et al., 2021). PTK2 (also named FAK) and PXN are signaling transducers that translate mechanical signals from transmembrane integrin to biochemical signals in the focal adhesion pathway; meanwhile, zyxin (coded by ZYX gene) acts as downstream actin cross-linking mediator that is tightly associated with the actomyosin apparatus (Horton et al., 2015). In our study, proteomic and enrichment analyses indicated that the focal adhesion signaling pathway was activated in VSMC after ICS-II treatment. Moreover, *in vitro* studies showed the enhanced protein expression levels of PTK2, PXN, and ZYX in the ICS-II group. Taken together, the current research suggests that the focal adhesion signaling pathway participates in ICS-II-induced VSMC contractile phenotype modulation.

Elastin (encoded by ELN) is the main extracellular matrix protein deposited on the arterial wall. Recent studies have indicated that elastin is not only required for ensuring the elasticity of the arterial wall but also promotes VSMC phenotypic modulation. Karnik et al. (2003) highlighted the critical role of ELN in inducing actin stress fiber organization and maintaining VSMC contractile phenotype (. Similarly, using an ELN deficiency model, Lin et al. (2021)showed that ELN knockout contributes to neointima hyperplasia and alters the VSMC phenotype (. In another study, the same group illustrated that the loss of elastin gene expression in VSMC causes discontinuous internal elastic lamina, which facilitates VSMC migration into the lumen, thus leading to neointima hyperplasia (Lin et al., 2019). Taken together, ELN, as an extracellular matrix component, is not only involved in the structural integrity of the vessel wall but also regulates VSMC phenotypic transformation in neointima formation and vascular remodeling.

Sophisticated interactive signaling pathways determined the phenotypic transition of VSMC and facilitated the development of cardiovascular diseases (Frismantiene et al., 2018). Liao et al. (2017) reported that the VEGF signaling pathway promoted VSMC proliferation and thus negatively correlated with the VSMC contractile phenotype. It is controversial that the VEGF signaling pathway was enriched in the up-regulated protein set after ICS-II intervention in our study. This may be attributed to the fact that endothelium proliferation is another major feature of the present animal model in addition to the VSMC phenotypic transition, and the VEGF signaling pathway is the major mechanism that promotes endothelium proliferation, although we scraped the endothelium during the sampling process. It is also noteworthy that the two genes enriched to the VEGF signaling pathway happened to be mainly involved in the focal adhesion pathway, which may lead to false-positive results of the enrichment analysis. The TGF-β signaling pathway was reported to be critical for VSMC phenotypic maintenance, and impairment of the TGF-β signaling pathway leads to the development of various cardiovascular diseases (Goumans et al., 2009). Consistent with this, we found that the TGF-β signaling pathway was enriched after ICS-II intervention. Conclusively, more in-depth studies are encouraged to decrypt the molecular basis and complicated signaling networks in the VSMC phenotypic transition process.

Increasing studies revealed that the VSMC phenotypic transition was driven by the metabolic switch, and VSMCs altered their metabolism to accommodate the bioenergetic demands during the phenotypic transition (Shi et al., 2020). Evidence showed that abnormal metabolic modulation of glucose, fatty acids, and amino acids, as well as metabolic crosstalk between VSMC and other cell types, played a role in the VSMC phenotypic transition during cardiovascular diseases (Shi et al., 2020). In our study, several metabolic signaling pathways were enriched after ICS-II intervention, such as oxidative phosphorylation, fatty acid metabolism, tryptophan, valine, leucine, and isoleucine metabolism, etc. Our results highlighted the underlying pivotal role of metabolism processes in the VSMC phenotypic transition during vascular remodeling; further studies are needed to emphasize the related metabolic signaling pathways during the VSMC phenotypic transition.

## Conclusion

In conclusion, the present study demonstrates that ICS-II, a plant flavonol glycoside, attenuates vascular remodeling by restoring the VSMC contractile phenotype *in vivo* and *in vitro*. The focal adhesion pathway participated in the pharmaceutical effects of ICS-II in VSMC phenotypic modulation. Our results highlighted the potential therapeutic efficacy of ICS-II in vascular remodeling diseases such as neointima formation and restenosis. Further investigations are needed to elucidate the underlying molecular mechanisms and signaling pathways related to VSMC phenotypic modulation after ICS-II intervention in vascular remodeling.

## Data Availability

The datasets presented in this study can be found in online repositories. The mass spectrometry proteomics data have been deposited to the ProteomeXchange Consortium (http://proteomecentral.proteomexchange.org) via the iProX partner repository with the dataset identifier PXD033724.
